# Structural and functional connectivity of motor circuits after perinatal stroke: A machine learning study

**DOI:** 10.1016/j.nicl.2020.102508

**Published:** 2020-11-19

**Authors:** Helen L. Carlson, Brandon T. Craig, Alicia J. Hilderley, Jacquie Hodge, Deepthi Rajashekar, Pauline Mouches, Nils D. Forkert, Adam Kirton

**Affiliations:** a Department of Pediatrics, Cumming School of Medicine, University of Calgary, Calgary, AB, Canada; b Calgary Pediatric Stroke Program, Alberta Children’s Hospital, Calgary, AB, Canada; c Alberta Children’s Hospital Research Institute, Calgary, AB, Canada; d Hotchkiss Brain Institute, University of Calgary, Calgary, AB, Canada; e Department of Radiology, University of Calgary, Calgary, AB, Canada; f Department of Clinical Neurosciences, University of Calgary, Calgary, AB, Canada

**Keywords:** Perinatal stroke, Pediatric, MRI, Machine learning, Resting state fMRI, Diffusion tractography

## Abstract

Developmental neuroplasticity allows young brains to adapt via experiences early in life and also to compensate after injury. Why certain individuals are more adaptable remains underexplored. Perinatal stroke is an ideal human model of neuroplasticity with focal lesions acquired near birth in a healthy brain. Machine learning can identify complex patterns in multi-dimensional datasets. We used machine learning to identify structural and functional connectivity biomarkers most predictive of motor function. Forty-nine children with perinatal stroke and 27 controls were studied. Functional connectivity was quantified by fluctuations in blood oxygen-level dependent (BOLD) signal between regions. White matter tractography of corticospinal tracts quantified structural connectivity. Motor function was assessed using validated bimanual and unimanual tests. RELIEFF feature selection and random forest regression models identified predictors of each motor outcome using neuroimaging and demographic features. Unilateral motor outcomes were predicted with highest accuracy (8/54 features r = 0.58, 11/54 features, r = 0.34) but bimanual function required more features (51/54 features, r = 0.38). Connectivity of both hemispheres had important roles as did cortical and subcortical regions. Lesion size, age at scan, and type of stroke were predictive but not highly ranked. Machine learning regression models may represent a powerful tool in identifying neuroimaging biomarkers associated with clinical motor function in perinatal stroke and may inform personalized targets for neuromodulation.

## Highlights

• Perinatal stroke is an ideal human model of developmental neuroplasticity.
• Machine learning can identify complex patterns in multi-modal neuroimaging data.
• Connectivity of both lesioned and non-lesioned hemispheres predict motor function.
• Connectivity of thalamus and basal ganglia structures also play a central role.
• Machine learning regression models may advance understanding of neuroplasticity.

## 1 Introduction

Developmental neuroplasticity refers to the brain’s ability to adapt based on experiences in early life. Young brains appear to have the remarkable ability of compensating for lost function after early injury (the Kennard Principle; Kennard, 1936). The specific neuroplastic mechanisms by which certain individuals are more adaptable than others remain poorly understood but has major implications for developing therapeutic interventions to improve outcomes. Perinatal stroke is an ideal human model for investigating such neuroplasticity with focal lesions acquired in an otherwise healthy brain around the time of birth (Kirton, 2013). Perinatal stroke affects millions, resulting in unilateral cerebral palsy and lifelong disabilities with no prevention strategies (Dunbar and Kirton, 2018).

Two main forms of perinatal stroke prevail (Dunbar and Kirton, 2018), arterial ischemic stroke (AIS) and periventricular venous infarction (PVI). AIS is brain infarction within an arterial territory, most commonly the middle cerebral artery, and encompasses both neonatal arterial ischemic stroke (NAIS) and arterial presumed perinatal ischemic stroke (APPIS)(Kirton et al., 2008). These differ only in timing of clinical presentation with NAIS diagnosed shortly after birth when seizures occur, while APPIS is typically diagnosed later in infancy when early motor asymmetry (*i.e.*, handedness) leads to imaging that confirms remote arterial stroke. Both types of AIS often result in large cortical and subcortical lesions (often affecting the basal ganglia) with recent precise 3D mapping studies in the neonate demonstrating the arterial territories affected (Stephan-Otto et al., 2017, Núñez et al., 2020). Conversely, PVI occurs *in utero* before 34 weeks gestation as a result of a germinal matrix hemorrhage with secondary medullary venous infarction. Damage from PVI is typically constrained to periventricular subcortical white matter, often including the corticospinal tract. With all types commonly injuring major components of the developing motor system, perinatal stroke accounts for most cases of hemiparetic or unilateral cerebral palsy. While most suffer such motor disability, additional lifelong morbidities include disorders of learning, intelligence, language, behaviour and epilepsy (Kirton, 2013, Dunbar and Kirton, 2018).

We and others have previously shown that various modalities of magnetic resonance imaging (MRI) can be used to explore how brain structure and function change as children with AIS and PVI (Hodge et al., 2017, Kuczynski et al., 2018, Saunders et al., 2019) or just PVI (Woodward et al., 2019) grow and adapt after their injury. The power of neuroimaging within this context lies in its ability to identify different but complementary biomarkers associated with clinical function. However, that each can only explore single or small numbers of metrics at one time remains a major limitation. Traditional analysis of neuroimaging datasets relies heavily on group-level statistics, which limits a translation of these findings for individual outcome predictions. Thus, a multimodal approach may prove fruitful for identifying more complex relationships between imaging biomarkers and function. The same conundrum exists for the study of interventional neuroplasticity where emerging neuromodulation trials in perinatal stroke are showing clinical promise (Kirton et al., 2016a, Kirton et al., 2017) and advanced imaging can shed light on possible mechanisms (Carlson et al., 2018). The neural targets for these trials are similarly informed by neuroimaging metrics (Hilderley et al., 2019) but struggle to understand which combination of measures best reflect individual level neurophysiology or personalized markers of responsiveness to treatment. There is therefore a need for robust, patient-specific methods of characterizing developmental and interventional plasticity.

Machine learning is gaining popularity in neuroimaging given its ability to identify complex patterns in multi-dimensional and multi-modal data which can then be used to make data-driven classifications and predictions for individual patients. The aim of the current work was to use machine learning to identify which multi-modal neuroimaging biomarkers of structural (SC) and functional connectivity (FC) are most predictive of clinical motor function in children with perinatal stroke. We hypothesized that type of stroke and the connectivity of the thalamus and basal ganglia structures (Lanciego et al., 2012) would play important roles in predicting motor function in addition to cortical primary motor areas given their central placement within motor circuits.

## 2 Materials and methods

### 2.1 Participants

Children with perinatal stroke were sampled from a large, population-based cohort of perinatal stroke cases occurring in southern Alberta and seen at the Alberta Children’s Hospital Pediatric Neurology Clinic (The Alberta Perinatal Stroke Project; Cole et al., 2017). Inclusion criteria were: 1) MRI confirmed unilateral perinatal stroke (AIS or PVI) according to established criteria (Kirton et al., 2008), 2) current age between 6 and 19 years and term birth, and 3) symptomatic hemiparetic cerebral palsy (HCP) [Pediatric Stroke Outcome Measure (PSOM) score > 0.5 (Kitchen et al., 2003) and perceived functional limitations by child and parent]. Exclusion criteria included MRI contraindications, clinical or imaging evidence of bilateral or additional brain injury, or unstable epilepsy.

For comparison, a group of typically developing control (TDC) participants similar in age (±1 year) and sex to the stroke group were recruited from a healthy controls recruitment database. These children were right-handed by self (or parent) report, had no MRI contraindications, neurodevelopmental or psychiatric conditions. Given that all control participants were right-handed, for group comparisons the dominant hemisphere (left) was compared to the non-lesioned hemisphere in the perinatal stroke participants and conversely, the non-dominant hemisphere (right) in controls was compared to the lesioned hemisphere in participants with stroke.

Informed parental consent and participant assent were obtained for all participants in accordance with the Declaration of Helsinki. This study was approved by the Research Ethics Board at the University of Calgary.

### 2.2 Imaging

MRIs were performed using a 3 Tesla General Electric (GE) MR750w scanner (GE Healthcare, Waukesha, WI) with a 32-channel head coil at the Alberta Children’ Hospital Research Imaging Suite. High-resolution T1-weighted fast spoiled gradient echo brain volume (FSPGR BRAVO) anatomical images were obtained in the axial plane [voxels = 1 mm isotropic, no skip, 166–225 slices, repetition time (TR)/echo time (TE)/inversion time (TI) = 8.5/3.2/600 ms, flip angle = 11°, duration ~ 5:00]. Diffusion-weighted images were also obtained axially for 32 non-collinear directions (voxels = 2.5 mm isotropic, 60 slices, b-value = 750 s/mm^2^, 3 b = 0 volumes, TR/TE = 11.5 s/69 ms, duration ~ 6:00). Resting-state functional MRI images were obtained while participants fixated on a centrally presented black cross and were told to think of “nothing in particular” (voxels = 3.6 mm isotropic, 36 axial slices, 150 volumes, TR/TE = 2000/30 ms, flip angle = 90°, duration ~ 5:00).

### 2.3 Structural connectivity

Each T1-weighted volume was segmented into grey matter (GM), white matter (WM), cortical spinal fluid (CSF), skull and skin using the automatic segmentation tool implemented in SPM12 (Statistical Parametric Mapping version 12, Wellcome Trust; https://www.fil.ion.ucl.ac.uk/spm/) using standard tissue probability maps. Stroke lesions were classified as CSF. FMRIB Software Library (FSL) *FIRST* algorithm (Patenaude et al., 2011) was used to segment subcortical structures. For each participant, tissue maps were then combined using the *5ttgen* command in MRtrix3 (Tournier et al., 2012) to create a 5-tissue-type image used to generate a GM-WM interface image for anatomically-constrained tractography (Smith et al., 2012). All T1 maps were then linearly co-registered to the diffusion scans using FSL’s *FLIRT* (Jenkinson et al., 2002). All steps were manually quality checked slice-by-slice to ensure accuracy.

**Fig. 1:**
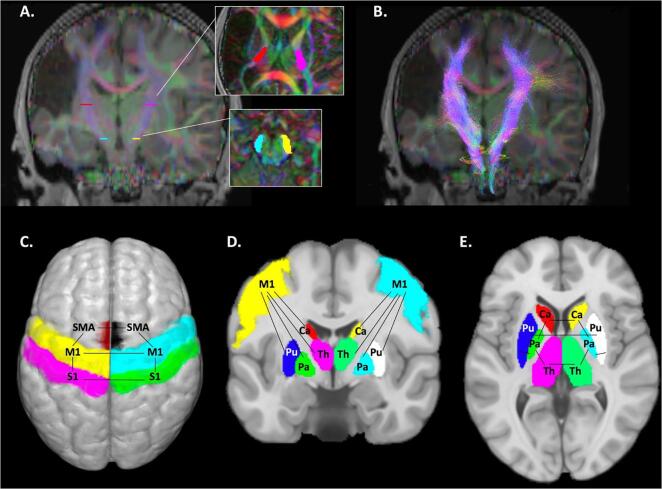
Neuroimaging features used to predict clinical motor function. Structural connectivity was measured via white matter tractography of the bilateral cortical spinal tract (CST). Regions-of-interest (ROIs) included the posterior limb of the internal capsule (red/pink ROIs) and the cerebral peduncles (yellow/cyan ROIs) (A). Underlying diffusion characteristics (mean FA, MD, RD, and AD) were extracted from CST masks overlaid on the diffusion maps (B). Functional connectivity was measured between cortical motor areas (C), cortico-subcortical areas (D), and within subcortical areas (E). For a complete list of features, see Table 1. FA – Fractional anisotropy; MD, RD, AD - Mean, radial, and axial diffusivity; M1 - Primary motor cortex, S1 - Primary sensory cortex, SMA - Supplementary motor area, Pu - Putamen, Ca - Caudate, Th - Thalamus, Pa - Pallidum. (For interpretation of the references to colour in this figure legend, the reader is referred to the web version of this article.)

FSL’s *eddy_correct* function was used to correct the diffusion datasets for eddy current distortions and for head motion realignment. In MRtrix3, constrained spherical deconvolution (CSD) was used to generate fibre orientation distribution (FOD) maps that code directionality of fibre populations and are robust to areas of crossing fibres (Tournier et al., 2012). The diffusion tensor was also used to generate whole brain maps of fractional anisotropy (FA), as well as mean (MD), radial (RD), and axial (AD) diffusivity. To isolate the bilateral cortical spinal tracts (CSTs), two regions of interest (ROIs) per hemisphere were manually drawn on an axial slice of the colour-coded FA map (Fig. 1A) to encompass the cerebral peduncle (cyan/yellow) and the posterior limb of the internal capsule (PLIC; red/pink) (Hodge et al., 2017, Kuczynski et al., 2018). Two exclusion ROIs were manually drawn on coronal slices to exclude streamlines from projecting anteriorly past the pre-central gyrus or posteriorly into the superior parietal lobule. Probabilistic tractography using the *tckgen* function reconstructed CSTs for lesioned and non-lesioned hemispheres (tracking algorithm: iFOD2, step size = 1 mm, angle threshold = 45°, FOD amplitude threshold = 0.05, 3000 streamlines, Fig. 1B). Exclusion ROIs were drawn to trim spurious fibres, which were most commonly corpus callosum and cerebellar projection fibres. Measures of WM microstructure (mean FA, MD, RD, and AD) for each lesioned and non-lesioned CST were extracted by overlaying a binary tract mask on microstructure maps of interest resulting in eight features representing WM structural connectivity (Table 1).

**Table 1:** List of demographic (5) and neuroimaging features (49) included in the initial feature selection.

Features	
**Demographic (5)**	**Cortico-subcortical FC (24)**
Age at scan (years)	Non-Les M1 – Non-Les Thalamus
Sex (male or female)	Non-Les M1 – Non-Les Caudate
Estimated lesion volume (ELV) (cc)	Non-Les M1 – Non-Les Pallidum
Side of stroke (right or left)	Non-Les M1 – Non-Les Putamen
Type of stroke (AIS or PVI)	Les M1 – Les Thalamus
**White matter SC (8)**	Les M1 – Les Caudate
Les CST FA, MD, AD, RD	Les M1 – Les Pallidum
Non-Les CST FA, MD, AD, RD	Les M1 – Les Putamen
**Cortical FC (7)**	Non-Les S1 – Non-Les Thalamus
Les M1 – Non-Les M1	Non-Les S1 – Non-Les Caudate
Les S1 – Non-Les S1	Non-Les S1 – Non Les Pallidum
Non-Les M1 – Non-Les S1	Non-Les S1 – Non Les Putamen
Les M1 – Les S1	Les S1 – Les Thalamus
Non-Les M1 – Non-Les SMA	Les S1 – Les Caudate
Les M1 – Les SMA	Les S1 – Les Pallidum
Non-Les SMA – Les SMA	Les S1 – Les Putamen
**Thalamus/basal ganglia FC (10)**	Non-Les SMA – Non-Les Thalamus
Les Thalamus – Non-Les Thalamus	Non-Les SMA – Non-Les Caudate
Les Caudate – Non-Les Caudate	Non-Les SMA – Non Les Pallidum
Les Pallidum – Non-Les Pallidum	Non-Les SMA – Non Les Putamen
Les Putamen – Non-Les Putamen	Les SMA – Les Thalamus
Non-Les Caudate – Non-Les Pallidum	Les SMA – Les Caudate
Non-Les Pallidum – Non-Les Thalamus	Les SMA – Les Pallidum
Non-Les Putamen – Non-Les Pallidum	Les SMA – Les Putamen
Les Caudate – Les Pallidum	
Les Pallidum – Les Thalamus	
Les Putamen – Les Pallidum	

Table Note: AIS – Arterial ischemic stroke, PVI – Periventricular venous infarction, cc – Cubic centimeters, Les – lesioned, SC – Structural connectivity, FC – Functional connectivity, CST – Cortical spinal tract, FA – Fractional anisotropy, MD, AD, RD – Mean, axial, and radial diffusivity, M1 – Primary motor cortex, S1 – Primary sensory cortex, SMA – Supplementary motor area. In TDC participants, non-lesioned refers to the dominant hemisphere (left) and lesioned refers to the non-dominant (right) hemisphere.

### 2.4 Functional connectivity

Resting-state functional MRI datasets were processed using the Connectivity Toolbox [CONN (Whitfield-Gabrieli and Nieto-Castanon, 2012) version 18.a] in MatLab (iMac version 2018a; Mathworks, Natick MA). Preprocessing utilized the validated CONN pipeline including slice timing correction, realignment, co-registration, calculation of head motion parameters, smoothing (using a 6 mm full-width half-maximum Gaussian kernel), and de-noising. Functional and structural images were normalized into standard Montreal Neurological Institute (MNI) space using the non-linear unified segmentation and normalization procedure in SPM12. Resulting normalized images were inspected slice-by-slice to ensure that the lesioned areas were appropriately segmented as CSF and normalized accurately. The Artifact Repair Toolbox (Mazaika et al., 2007) was used to identify head motion and other outliers using thresholds for global mean signal (z > 5) and/or translational movement (>0.9 mm). Identified volumes were de-weighted in the first-level general linear model (GLM) as were time courses of CSF and WM signal.

Functional connectivity among sensorimotor network ROIs was calculated via temporal cross correlations of signal fluctuations over time represented as Fisher-transformed bivariate Pearson correlation coefficients. ROI-ROI pairs used as FC features in the predictive model were selected based on established models of direct and indirect motor circuit anatomy that form cortico-basal ganglia-thalamo-cortical loops facilitating voluntary movements (Lanciego et al., 2012, Simonyan, 2019). ROIs were defined using the validated Harvard-Oxford atlas provided in CONN. Cortical ROIs included bilateral primary motor (M1), sensory (S1), and supplementary motor (SMA) areas. Subcortical ROIs included bilateral thalamus and basal ganglia (putamen, caudate, and pallidum). Forty-one functional connections of interest were identified and are listed in Table 1, a subset of which are illustrated in Fig. 1C-E.

### 2.5 Lesion volumes

To estimate lesion volumes, whole brain volumes of GM, WM, and CSF [in cubic centimetres (cc)] were extracted from the SPM12 segmentation masks generated during the functional connectivity processing pipeline (described above). Estimated total intracranial volume (eTIV) was calculated as the sum of the GM, WM, and CSF mask volumes. Estimated lesion volumes (ELV in cc), corrected for intracranial volume, were therefore calculated as described below for each patient (where larger numbers represent larger relative lesions):

$$\mathrm{ELV}=1-\left[\frac{\left(GM+WM\right)}{\mathrm{eTIV}}\right]$$

In AIS patients, lesions reflecting tissue loss are typically filled with CSF and thus larger volumes of CSF present after AIS would be captured in a higher ELV. In PVI patients, dilatation of the ventricle in the lesioned hemisphere is reflective of the size of the periventricular WM lesion and these larger volumes of ventricle CSF would be quantified by ELV calculations. Brain images for our PVI sample were reviewed slice-by-slice and no cystic lesions were identified beyond the ventricular border, removing the need for any additional segmentation to capture total lesion volume.

### 2.6 Clinical motor function

Clinical motor function was assessed using validated bimanual [Assisting Hand Assessment (AHA) (Krumlinde-sundholm and Eliasson, 2003)] and unimanual [Box and Blocks Test (BBT) (Mathiowetz et al., 1985)] tests. The AHA is a 22-item assessment tool that measures spontaneous bimanual hand function in children with unilateral motor impairments. It has the advantage of using real-world activities to measure spontaneous bimanual motor function rather than testing the affected hand in isolation. Motor outcomes range from 0 (hand is not used at all) to 100 (normal motor function)(Krumlinde-sundholm and Eliasson, 2003). The BBT is a performance-based measure of upper limb dexterity in which participants move a series of blocks from one container into another as quickly as possible. Two BBT scores were collected, one for the stroke-affected hand (BBTA) and one for the unaffected hand (BBTU) by counting the number of blocks successfully transferred in 60 s (Mathiowetz et al., 1985). For all three measures, higher scores indicate better performance.

### 2.7 Feature selection & regression model

It is common knowledge that the performance of a predictive machine learning model can be improved by selecting a subset of input features that not only explain most of the variance in the outcome score but also contain no redundant information. This feature selection step is performed before the regression models are built in order to reduce the multicollinearity among features being tested thus reducing the redundancies in the data. In this work, the Relief feature selection algorithm for regression models (RRELIEFF) was employed to rank the 54 features available (49 imaging features and five demographic features: age, sex, ELV, side, and type of stroke; Table 1) with respect to their relevance for each of the three outcome scores (AHA, BBTA, and BBTU) and three patient groups (AIS, PVI, AIS + PVI combined) independently. Briefly, the RRELIEFF algorithm determines weights for each feature based on the values of the attributes amongst the nearest neighbors of a randomly selected patient sample (Kononenko et al., 1997). The input features were normalized (min–max) prior to estimating the feature ranks.

After feature ranking, the random forest machine learning algorithm was used to model the regression problem for each outcome score separately. More precisely, a random decision tree forest with 100 decision trees was trained on a random subset of data such that nodes in the higher branches of each decision tree maximally differentiate the selected training samples. For a given test sample, the predictions from all decision trees in the random forest are averaged to determine the final prediction of the outcome scores. The random decision tree forest model was used in this work due to its ability to combine various input feature types for solving regression problems and reduced risk of overfitting compared to other machine learning models (Polikar, 2006, Sagi and Rokach, 2018, Vercio et al., 2020). Additionally, random forests are known to deal well with high-dimensional data while also being robust to outliers and non-linear data. Finally, the random forest decision tree model does not require many hyperparameters to tune, further decreasing the risk of overfitting. Additionally, RRELIEFF feature ranking was used as it provides a ranking of features based on their relative importance in predicting outcome scores. This ranking allows us to determine which demographic and/or connectivity patterns were most highly predictive of motor function, thus informing rehabilitation strategies and identifying targets for non-invasive brain stimulation.

In order to prevent double dipping, the feature selection and random forest regression model were implemented within a nested leave-one-out-patient cross validation scheme so that no information from the patient to be tested in each iteration was used for feature ranking or training of the random forest regression model. Owing to the small sample size, leave-one-out cross validation was employed to reduce the likelihood of overfitting the regression models (Kuhn and Johnson, 2013).

The selection of the optimal set of features for each outcome score was done iteratively by removing the least informative feature from the ranked features until only one feature was left for training of the regression model ensuring that redundant and non-informative features, which can downgrade the accuracy of the regression model, are eliminated. The optimal feature subset for each outcome score was identified by determining the model resulting in the lowest root mean squared error (RMSE) comparing the true outcome scores with the predicted outcome scores from the leave-one-out cross validations. The accuracy of these optimal regression models were further quantified using coefficient of determination (R^2^) values that capture the amount of variance in the dependent variable that can be accounted for by the independent variables (features) in the regression model as well as the corresponding correlation values.

A total of nine regression models investigated predictive features for three motor tasks (AHA, BBTA, BBTU) in three patient groupings (AIS, PVI, AIS + PVI combined). Each of the two stroke types were investigated separately so as to determine disease-specific features predictive of function. The AIS + PVI combined group was used to investigate features predictive of function after early injury to motor circuits (regardless of mechanism) while maximizing sample size.

### 2.8 Statistical analysis

Distribution normality was tested using Shapiro-Wilk. Differences between patient groups (AIS, PVI) were subsequently examined using two-tailed independent sample t-tests for ELV and motor outcomes (AHA, BBTU, BBTA). Kruskal-Wallis tested for differences in age between the three groups. Pearson’s Chi-Square test examined differences in sex and side of stroke between groups. For neuroimaging variables, relationships with age were measured using Pearson’s r or Spearman’s rho correlations as appropriate. Differences in neuroimaging variables between groups (AIS, PVI, TDC) were tested using either one-way Analysis of Variance (ANOVA) followed by Bonferroni-corrected post-hoc tests (if normally distributed) or Kruskal-Wallis followed by Bonferroni-corrected pairwise comparisons (if not normally distributed). A threshold of α = 0.05 was used to determine statistical significance. Cohen’s d quantified effect sizes. Statistics were performed using SPSS version 26 (IBM, Armonk, USA).

### 2.9 Data availability

Data will be provided upon reasonable request.

## 3 Results

### 3.1 Participants

Fifty-five children with perinatal stroke and 27 controls were initially recruited. Of the children with stroke, six were subsequently excluded for the following reasons: no measurable CST in the lesioned hemisphere (N = 3), large amplitude head motion during MRI (N = 2), or a very large lesion that precluded accurate tissue segmentation (N = 1) leading to a final sample of 49 participants with stroke. Group comparisons for neuroimaging features were performed using 27 controls, 22 AIS and 27 PVI (Table 2). For the regression analyses, TDC children were excluded since the AHA is specialized for quantifying unilateral motor impairments in patients and the remaining tasks (BBTA and BBTU) were not available for the control sample. A further patient was excluded from the BBTA analyses due to a missing score. Thus, the samples studied using regression models were 48 patients for the BBTA analysis and 49 patients for the BBTU and AHA analyses.

**Table 2:** Demographic characteristics and motor function of patient groups.

**Category**	**TDC (N = 27)**	**AIS (N = 22)**	**PVI (N=27)**	**Total Stroke (N=49)**
Age	12.9 (3.4) [6.5–18] y	13.0 (3.8) [6.6-19] y	11.3 (3.3) [6.7–19.7] y	12.1 (3.5) [6.6–19.7] y
Sex	Male N = 14 Female N = 13	Male N = 15 Female N = 7	Male N = 17 Female N = 10	Male N = 32 Female N = 17
Side	–	Left N = 15 Right N = 7	Left N = 16 Right N = 11	Left N = 31 Right N = 18
ELV	–	0.44 (0.1) [0.3–0.6] cc	0.34 (0.1) [0.2–0.5] cc	0.38 (0.1) [0.2–0.6] cc
BBTA	–	24.5 (15.1) [3–58]	30.5 (10.1) [14–50]	27.9 (12.8) [3–58]
BBTU	–	52.3 (9.4) [38–71]	52.6 (13.5) [19–84]	52.63 (11.7) [19–84]
AHA	–	58.9 (17.7) [29–100]	65.8 (12.8) [47–100]	62.0 (16.0) [27–100]

Table note: BBT - Box and Blocks Test (BBTA – Affected; BBTU - Unaffected hand), AHA – Assisting Hand Assessment, SD – Standard deviation, TDC – Typically Developing Controls, PVI – Periventricular venous infarction, Side – MRI confirmed diagnosis of stroke and side, ELV – Estimated lesion volume in cubic centimetres (cc). Values are expressed as mean (SD) [range] unless otherwise specified.

Demographics were comparable between groups for all demographics (Table 2). Specifically, age at scan [H_(2)_ = 4.7, p = 0.095], sex [χ^2^_(2)_ = 1.46, p = 0.48] and side of stroke [χ^2^_(1)_ = 0.42, p = 0.52] did not differ among the groups. The AIS group showed significantly higher ELV compared to PVI (t_(48)_ = 5.53, p < 0.0001, d = 1.0). Motor function was also comparable between the two patient groups; AIS scores were largely lower than PVI reflecting more motor impairment but these differences were not statistically significant (AHA: t_(47)_ = -1.87, p = 0.07, d = 0.61; BBTA: t_(46)_ = -1.64, p = 0.11, d = 0.47; BBTU: t_(47)_ = -0.18, p = 0.86, d = 0.03). Age was not related to neuroimaging variables and was thus not factored out in the participant group comparisons though was subsequently included in the regression models as a proxy for “time since stroke”.

### 3.2 Group differences in neuroimaging variables

**Fig. 2:**
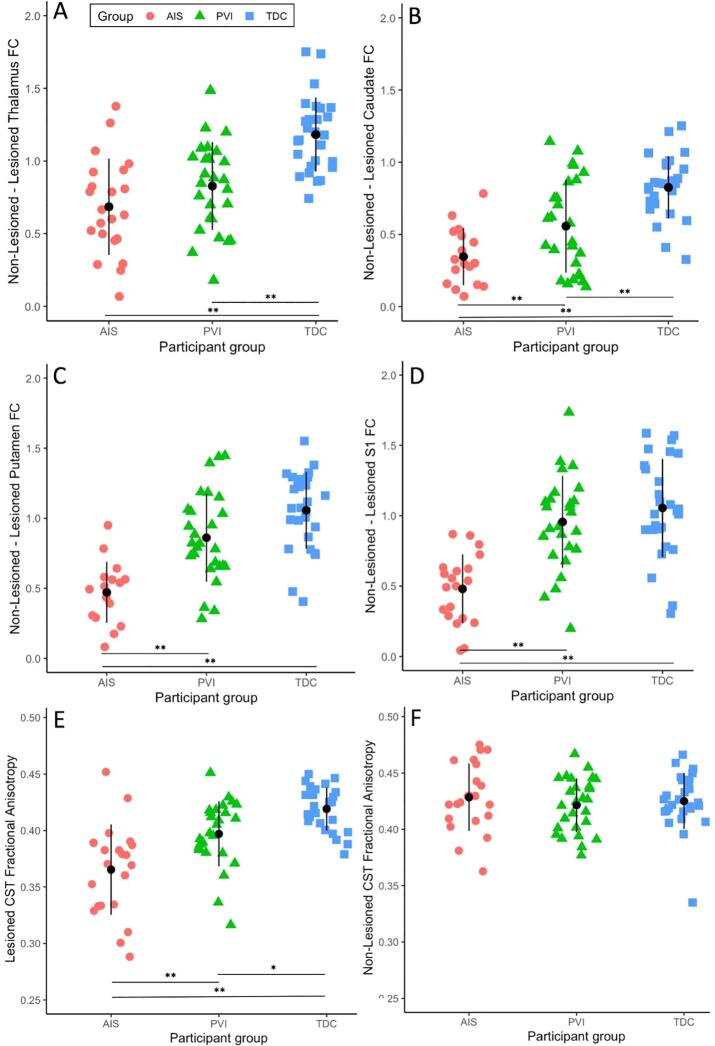
Functional and structural connectivity values by participant group. Inter-hemispheric functional connectivity (FC) for stroke groups was significantly lower compared to TDC between the non-lesioned and lesioned (A) thalamus, (B) caudate, (C) putamen and (D) primary sensory cortex (S1). Fractional Anisotropy was lower for both AIS and PVI groups compared to TDC for the (E) lesioned but not the (F) non-lesioned corticospinal tract (CST). Black symbols represent the mean and vertical lines represent standard deviation. ** p ≤ 0.001, * p < 0.05. AIS – Arterial ischemic stroke, PVI – periventricular venous infarction, TDC – typically developing controls.

Resting state functional connectivity was largely similar between the TDC and PVI groups, however the AIS group showed lower functional connectivity compared to both TDC and PVI for cortical features (Table 3). Specifically, functional connectivity between lesioned and non-lesioned M1, S1 and SMA was much lower for the AIS group compared to PVI and TDC. Inter-hemispheric connectivity between basal ganglia structures (caudate, pallidum, putamen) as well as the thalamus showed a similar pattern among groups (Fig. 2), however intra-hemispheric connectivity within these regions was not different between groups. Connectivity between cortical and subcortical structures showed few differences between AIS, PVI and TDC with the exception of the lesioned-side putamen which showed lower connectivity with M1, S1 and SMA for AIS compared to both PVI and TDC.

**Table 3:** Neuroimaging feature values by group with statistical contrasts between groups.

Neuroimaging Feature	Participant Group			Contrasts		
Mean (SD) [range]	TDC	AIS	PVI	AIS vs TDC	PVI vs TDC	AIS vs PVI
**White matter SC features**						
Les CST FA	0.42 (0.02) [0.38-0.45]	0.36 (0.04) [0.29-0.45]	0.40 (0.03) [0.32-0.45]	p < 0.0001	p = 0.024	p = 0.001
Les CST MD	8.36 (0.32) [7.54-8.91]	9.54 (0.63) [8.43-10.7]	9.01 (1.1) [8.1-14.4]	p < 0.0001	p = 0.003	p = 0.007
Les CST AD	12.3 (0.38) [11.3-13.0]	13.3 (0.65) [12.1-15.0]	12.7 (0.45) [11.7-13.5]	p < 0.0001	p = 0.02	p < 0.0001
Les CST RD	6.37 (0.33) [5.65-7.04]	7.64 (0.70) [6.33-9.04]	6.93 (0.56) [6.15-8.53]	p < 0.0001	p = 0.002	p = 0.008
Non-Les CST FA	0.42 (0.03) [0.34-0.47]	0.43 (0.03) [0.36-0.48]	0.42 (0.02) [0.38-0.47]	ns	ns	ns
Non-Les CST MD	8.23 (0.33) [7.81-9.45]	8.16 (0.32) [7.64-9.07]	8.30 (0.29) [7.67-8.95]	ns	ns	ns
Non-Les CST AD	12.2 (0.30) [11.8-12.9]	12.0 (0.90) [8.18-13.1]	12.3 (0.30) [11.6-12.9]	ns	ns	ns
Non-Les CST RD	6.21 (0.39) [5.57-7.74]	6.16 (0.39) [5.52-7.06]	6.33 (0.34) [5.64-6.99]	ns	ns	ns

**Cortical FC Features**						
Les M1 – Non-Les M1	0.88 (0.35) [−0.11-1.48]	0.37 (0.33) [−0.42-0.99]	0.96 (0.31) [0.04-1.42]	p < 0.0001	ns	p < 0.0001
Les S1 – Non-Les S1	1.05 (0.35) [0.31-1.59]	0.42 (0.30) [−0.25-0.87]	0.96 (0.33) [0.20-1.73]	p < 0.0001	ns	p < 0.0001
Non-Les M1 – Non-Les S1	0.76 (0.33) [0.00-1.20]	0.90 (0.27) [0.44-1.51]	0.85 (0.26) [0.32-1.37]	ns	ns	ns
Les M1 – Les S1	0.77 (0.38) [0.20-1.52]	0.73 (0.29) [0.26-1.23]	0.91 (0.29) [0.38-1.58]	ns	ns	ns
Non-Les M1 – Non-Les SMA	0.76 (0.15) [0.47-1.04]	0.48 (0.32) [−0.05-1.22]	0.85 (0.26) [0.23-1.29]	p = 0.001	ns	p < 0.0001
Les M1 – Les SMA	0.78 (0.26) [0.18-1.25]	0.40 (0.26) [−0.04-0.99]	0.75 (0.24) [0.39-1.20]	p < 0.0001	ns	p < 0.0001
Non-Les SMA – Les SMA	1.29 (0.29) [0.56-1.90]	0.99 (0.35) [0.36-1.52]	1.28 (0.28) [0.63-1.76]	p = 0.003	ns	p = 0.004

**Thalamus/basal ganglia FC features**						
Les Thalamus – Non-Les Thalamus	1.18 (0.26) [0.74-1.75]	0.68 (0.33) [0.07-1.38]	0.83 (0.30) [0.18-1.49]	p < 0.0001	p < 0.0001	ns
Les Caudate – Non-Les Caudate	0.83 (0.22) [0.33-1.25]	0.23 (0.28) [−0.42-0.78]	0.54 (0.33) [−0.1-1.14]	p < 0.0001	p = 0.001	p = 0.001
Les Pallidum – Non-Les Pallidum	1.01 (0.20) [0.62-1.43]	0.27 (0.35) [−0.35-0.97]	0.78 (0.31) [0.13-1.35]	p < 0.0001	p = 0.011	p < 0.0001
Les Putamen – Non-Les Putamen	1.06 (0.27) [0.41-1.55]	0.34 (0.32) [-0.33-0.95]	0.86 (0.31) [0.28-1.45]	p < 0.0001	ns	p < 0.0001
Non-Les Caudate – Non-Les Pallidum	0.15 (0.20) [−0.25-0.50]	0.14 (0.19) [−0.47-0.36]	0.23 (0.22) [-0.13-0.77]	ns	ns	ns
Non-Les Pallidum – Non-Les Thalamus	0.36 (0.26) [−0.13-0.78]	0.29 (0.24) [−0.10-0.85]	0.38 (0.23) [−0.09-0.82]	ns	ns	ns
Non-Les Putamen – Non-Les Pallidum	1.06 (0.22) [0.63-1.51]	0.91 (0.31) [0.31-1.50]	1.02 (0.23) [0.53-1.48]	ns	ns	ns
Les Caudate – Les Pallidum	0.11 (0.17) [−0.25-0.56]	0.04 (0.23) [−0.56-0.65]	0.02 (0.22) [−0.32-0.58]	ns	ns	ns
Les Pallidum – Les Thalamus	0.35 (0.28) [−0.10-0.91]	0.33 (0.35) [−0.30-0.72]	0.29 (0.27) [−0.13-0.84]	ns	ns	ns
Les Putamen – Les Pallidum	0.95 (0.21) [0.55-1.62]	0.79 (0.26) [0.32-1.35]	0.91 (0.26) [0.32-1.47]	ns	ns	ns

**Cortico-subcortical FC features**						
Non-Les M1 – Non-Les Thalamus	0.04 (0.19) [−0.29-0.38]	0.08 (0.24) [−0.37-0.48]	0.10 (0.21) [−0.25-0.42]	ns	ns	ns
Non-Les M1 – Non-Les Caudate	-0.06 (0.20) [−0.41-0.55]	-0.02 (0.24) [−0.50-0.35]	0.00 (0.20) [−0.34-0.41]	ns	ns	ns
Non-Les M1 – Non-Les Pallidum	0.18 (0.21) [−0.26-0.54]	0.20 (0.21) [−0.12-0.74]	0.20 (0.26) [−0.35-0.72]	ns	ns	ns
Non-Les M1 – Non-Les Putamen	0.18 (0.16) [−0.18-0.52]	0.24 (0.27) [−0.13-0.68]	0.26 (0.22) [−0.21-0.66]	ns	ns	ns
Les M1 – Les Thalamus	0.00 (0.21) [−0.43-0.42]	-0.12 (0.22) [-0.47-0.31]	0.01 (0.18) [−0.37-0.39]	ns	ns	ns
Les M1 – Les Caudate	-0.14 (0.16) [−0.39-0.19]	-0.12 (0.21) [−0.46-0.30]	-0.04 (0.16) [−0.38-0.38]	ns	ns	ns
Les M1 – Les Pallidum	0.08 (0.19) [−0.21-0.51]	-0.04 (0.21) [−0.49-0.31]	0.10 (0.24) [−0.35-0.50]	ns	ns	ns
Les M1 – Les Putamen	0.16 (0.21) [−0.32-0.56]	-0.03 (0.17) [−0.33-0.28]	0.19 (0.24) [−0.28-0.53]	p = 0.007	ns	p = 0.002
Non-Les S1 – Non-Les Thalamus	0.04 (0.19) [−0.38-0.47]	0.10 (0.21) [−0.19-0.56]	0.07 (0.20) [−0.36-0.50]	ns	ns	ns
Non-Les S1 – Non-Les Caudate	-0.11 (0.18) [−0.42-0.22]	-0.05 (0.19) [−0.33-0.36]	-0.08 (0.19) [−0.49-0.25]	ns	ns	ns
Non-Les S1 – Non Les Pallidum	0.01 (0.16) [−0.26-0.28]	0.10 (0.19) [−0.18-0.56]	0.10 (0.26) [−0.46-0.73]	ns	ns	ns
Non-Les S1 – Non Les Putamen	0.10 (0.15) [−0.32-0.34]	0.17 (0.21) [−0.17-0.62]	0.19 (0.22) [−0.22-0.66]	ns	ns	ns
Les S1 – Les Thalamus	0.02 (0.23) [−0.47-0.44]	-0.10 (0.23) [−0.44-0.28]	0.01 (0.18) [−0.39-0.27]	ns	ns	ns
Les S1 – Les Caudate	-0.10 (0.18) [−0.39-0.29]	-0.17 (0.23) [−0.58-0.47]	-0.03 (0.20) [−0.44-0.31]	ns	ns	ns
Les S1 – Les Pallidum	0.00 (0.16) [−0.36-0.33]	-0.06 (0.24) [−0.46-0.43]	0.01 (0.19) [−0.54-0.41]	ns	ns	ns
Les S1 – Les Putamen	0.12 (0.17) [−0.13-0.60]	-0.10 (0.19) [−0.40-0.36]	0.10 (0.23) [−0.41-0.56]	p < 0.0001	ns	p = 0.002
Non-Les SMA – Non-Les Thalamus	0.10 (0.22) [−0.39-0.45]	-0.09 (0.21) [−0.47-0.24]	0.11 (0.23) [−0.35-0.60]	p = 0.015	ns	p = 0.007
Non-Les SMA – Non-Les Caudate	0.03 (0.19) [−0.40-0.64]	0.01 (0.22) [−0.77-0.35]	0.06 (0.26) [−0.32-0.81]	ns	ns	ns
Non-Les SMA – Non Les Pallidum	0.15 (0.21) [−0.40-0.57]	0.03 (0.17) [−0.29-0.41]	0.21 (0.26) [−0.30-0.68]	ns	ns	p = 0.016
Non-Les SMA – Non Les Putamen	0.21 (0.18) [−0.26-0.49]	0.08 (0.24) [−0.50-0.60]	0.28 (0.24) [−0.20-0.73]	ns	ns	p = 0.005
Les SMA – Les Thalamus	0.11 (0.22) [−0.37-0.47]	-0.09 (0.22) [−0.46-0.25]	-0.02 (0.21) [−0.39-0.58]	p = 0.009	ns	ns
Les SMA – Les Caudate	-0.04 (0.17) [−0.38-0.25]	-0.04 (0.19) [−0.39-0.30]	-0.05 (0.19) [−0.36-0.56]	ns	ns	ns
Les SMA – Les Pallidum	0.14 (0.20) [−0.31-0.45]	-0.03 (0.17) [−0.32-0.35]	0.12 (0.26) [−0.54-0.63]	p = 0.036	ns	ns
Les SMA – Les Putamen	0.25 (0.23) [−0.39-0.67]	-0.02 (0.21) [−0.41-0.35]	0.22 (0.31) [−0.41-0.97]	p = 0.002	ns	p = 0.006

Table Note: AIS – Arterial ischemic stroke, PVI – Periventricular venous infarction, TDC – Typically Developing Controls, Les – lesioned, SC – Structural connectivity, FC – Functional connectivity, CST – Cortical spinal tract, FA – Fractional anisotropy, MD, AD, RD – Mean, axial, and radial diffusivity (x10^-4^), M1 – Primary motor cortex, S1 – Primary sensory cortex, SMA – Supplementary motor area. In TDC participants, non-lesioned refers to the dominant hemisphere (left) and lesioned refers to the non-dominant (right) hemisphere. ns – non-significant. Reported p-values are Bonferroni corrected.

Examinations of white matter microstructural features of the lesioned CST showed significantly lower FA and higher MD, AD and RD for both the AIS and PVI groups compared to TDC (Fig. 2). By contrast, white matter features for the non-lesioned CST were not different between the three groups.

### 3.3 Regression models

#### 3.3.1 AIS models

**Fig. 3:**
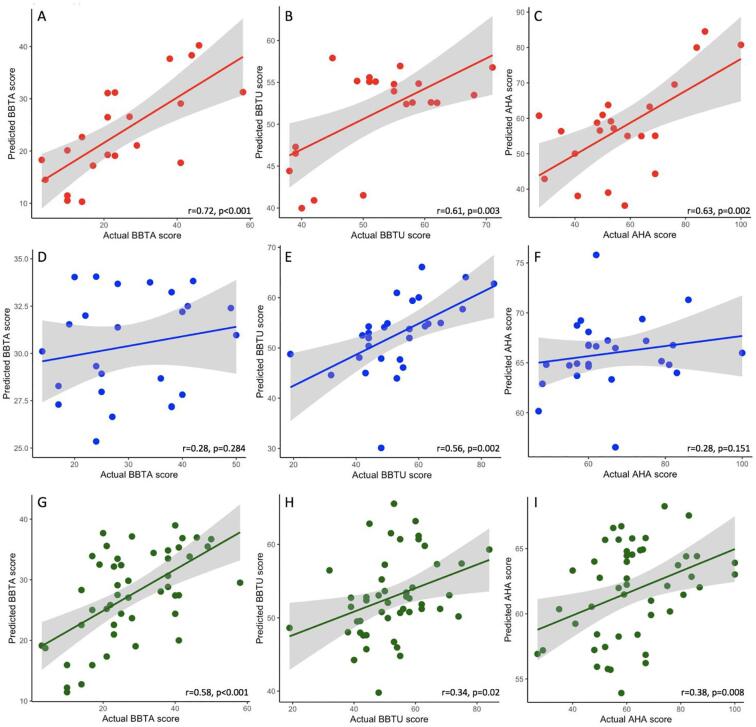
Regression models represented as scatterplots illustrating relationships between predicted and actual scores for motor outcomes on the BBTA, BBTU and AHA for (A-C) AIS, (D-F) PVI and (G-I) AIS + PVI groups combined. Shaded areas represent 95% confidence intervals of the regression line. AIS – Arterial ischemic stroke, PVI – periventricular infarction, TDC – Typically developing controls, BBT – Box and Blocks Test (BBTA - Affected hand, BBTU – Unaffected hand), AHA – Assisting Hand Assessment.

For the AIS group, individual predicted patient scores correlated highly with actual obtained scores (Fig. 3) for the three motor tasks (BBTA r = 0.719, p < 0.001; BBTU r = 0.610, p = 0.003; AHA r = 0.632, p = 0.002). Root mean squared error was lowest for the BBTU (RMSE BBTU = 7.35) and highest for AHA (RMSE BBTA = 10.42; RMSE AHA = 14.15).

Regression models for the AIS group required relatively few features (Table 4) for optimal prediction of function. The optimum prediction model for BBTA contained 6/54 features, accounted for 51.7% of the variance (as measured by R^2^) and consisted of both functional and structural connectivity measures as well as ELV. For BBTU, 5/54 features were required to account for 37.2% of variance. These features were solely measures of cortical inter-hemispheric and cortico-subcortical functional connectivity. The AHA model required only 2/54 cortico-subcortical (basal ganglia) functional connectivity features to explain 40% of variance.

**Table 4:** Most highly predictive features for BBTA, BBTU and AHA for the AIS group.

AIS group								
BBTA (R^2^ = 0.517 for 6 features)			BBTU (R^2^ = 0.372 for 5 features)			AHA (R^2^ = 0.400 for 2 features)		
Rank	Feature	Mod	Rank	Feature	Mod	Rank	Feature	Mod
1	Les S1 – Les Pallidum	FC	1	Non-Les SMA – Les SMA	FC	1	Les S1 – Les Pallidum	FC
2	Non-Les SMA – Les SMA	FC	2	Non-Les S1 – Non-Les Putamen	FC	2	Les SMA – Les Thalamus	FC
3	Les Putamen – Non-Les Putamen	FC	3	Les Putamen – Non-Les Putamen	FC			
4	Les CST RD	SC	4	Non-Les M1 – Non-Les Pallidum	FC			
5	Non-Les M1 – Non-Les SMA	FC	5	Les S1 – Non-Les S1	FC			
6	ELV	DE						

Table note: AIS – Arterial ischemic stroke, BBT – Box and Blocks Test (BBTA - Affected hand, BBTU – Unaffected hand), AHA – Assisting Hand Assessment, Mod – Modality, FC – Functional connectivity, SC – Structural connectivity, DE - Demographic, Les – Lesioned, CST – Cortical spinal tract, FA – Fractional anisotropy, MD - Mean diffusivity, M1 – Primary motor cortex, S1 – Primary sensory cortex, SMA – Supplementary motor area.

#### 3.3.2 PVI models

For the PVI group, correlations between actual and predicted motor scores were lower than for the AIS group (BBTA r = 0.284, p = 0.15; BBTU r = 0.559, p = 0.002; AHA r = 0.184, p = 0.36; Fig. 3). Error terms showed a similar pattern across motor tasks (RMSE BBTA = 9.53; RMSE BBTU = 10.86; RMSE AHA = 12.40) as in AIS.

Optimal prediction models for BBTA and AHA required more features compared to the AIS group (44/54 and 28/54 respectively), accounted for<10% of variance (Table 5) and was composed of both structural and functional connectivity features. By contrast, BBTU required only 3/54 features (explaining 31.2% of variance), two of which involved subcortical structures (putamen and thalamus).

**Table 5:** Most highly predictive features for BBTA, BBTU and AHA for the PVI group.

PVI group								
BBTA (R^2^ = 0.081 for 44 features, top 20 shown)			BBTU (R^2^ = 0.312 for 3 features)			AHA (R^2^ = 0.034 for 28 features, top 20 shown)		
Rank	Feature	Mod	Rank	Feature	Mod	Rank	Feature	Mod
1	Les S1 – Non-Les S1	FC	1	Les Putamen – Non-Les Putamen	FC	1	Les S1 – Non-Les S1	FC
2	Les CST AD	SC	2	Age at scan	DE	2	Non-Les M1 – Non-Les Thalamus	FC
3	Non-Les CST FA	SC	3	Les M1 – Les Thalamus	FC	3	Les S1 – Les Putamen	FC
4	Non-Les M1 – Non-Les SMA	FC				4	Les CST AD	SC
5	Age at scan	DE				5	Les Putamen – Non-Les Putamen	FC
6	Non-Les S1 – Non-Les Putamen	FC				6	Les M1 – Les Putamen	FC
7	Non-Les S1 – Non-Les Thalamus	FC				7	Les Pallidum – Les Thalamus	FC
8	Les M1 – Les S1	FC				8	Les M1 – Les Caudate	FC
9	Les M1 – Les Caudate	FC				9	Age at scan	DE
10	Non-Les CST AD	SC				10	Sex	DE
11	Les S1 – Les Putamen	FC				11	Les S1 – Les Pallidum	FC
12	Non-Les M1 – Non-Les Thalamus	FC				12	Non-Les M1 – Non-Les Caudate	FC
13	Non-Les CST RD	SC				13	Non-Les CST AD	SC
14	Les SMA – Les Thalamus	FC				14	Non-Les S1 – Non-Les Putamen	FC
15	Les M1 – Les Putamen	FC				15	Les Pallidum – Non-Les Pallidum	FC
16	Les Putamen – Non-Les Putamen	FC				16	Les Thalamus – Non-Les Thalamus	FC
17	Les CST FA	SC				17	Non-Les M1 – Non-Les SMA	FC
18	Les Putamen – Les Pallidum	FC				18	Les S1 – Les Thalamus	FC
19	Les S1 – Les Pallidum	FC				19	Non-Les S1 – Non-Les Thalamus	FC
20	Les Thalamus – Non-Les Thalamus	FC				20	Non-Les M1 – Non-Les Pallidum	FC

Table note: PVI – Periventricular venous infarction. BBT – Box and Blocks Test (BBTA - Affected hand, BBTU – Unaffected hand), AHA – Assisting Hand Assessment, Mod – MRI modality, FC – Functional connectivity, SC – Structural connectivity, DE - Demographic, Les – Lesioned, CST – Cortical spinal tract, FA – Fractional anisotropy, MD - Mean diffusivity, AD – Axial diffusivity, RD – Radial diffusivity, M1 – Primary motor cortex, S1 – Primary sensory cortex, SMA – Supplementary motor area.

#### 3.3.3 AIS + PVI combined models

For the AIS + PVI combined group, the correlation between actual and predicted scores for the BBTA (r = 0.582, p < 0.001) was higher than that for the BBTU (r = 0.343, p = 0.02) and the AHA (r = 0.376, p = 0.008; Fig. 3). The root mean squared error terms (RMSE) were comparable for the BBTA model (RMSE = 10.27) and the BBTU (RMSE = 10.98) but lower than the AHA model (RMSE = 14.92).

The optimal BBTA prediction model required 8/54 features (Table 6), which accounted for 33.8% of variance. Microstructure (radial diffusivity) of the lesioned CST was ranked highest in the BBTA model. Cortical-subcortical functional connectivity between S1 and pallidum in the lesioned hemisphere was the second-most highly ranked feature. Inter-hemispheric FC between non-lesioned M1 and supplementary motor area (SMA) as well as inter-hemispheric FC between bilateral SMA were also ranked as highly predictive of BBTA motor outcomes within the model. Other highly ranked features (2 out of 8 features) included FC between subcortical structures (putamen) and FC from non-lesioned M1 to non-lesioned thalamus. Age at scan (ranked 5/8) and ELV (ranked 6/8) were also included as predictive features for BBTA scores.

**Table 6:** Most highly predictive features for BBTA, BBTU and AHA for the AIS + PVI combined group.

BBTA (R^2^ = 0.338 for 8 features)			BBTU (R^2^ = 0.118 for 11 features)			AHA (R^2^ = 0.142 for 51 features, top 20 shown)		
Rank	Feature	Mod	Rank	Feature	Mod	Rank	Feature	Mod
1	Les CST RD	SC	1	Les Putamen – Non-Les Putamen	FC	1	Les S1 – Les Pallidum	FC
2	Les S1 – Les Pallidum	FC	2	Les Pallidum – Non-Les Pallidum	FC	2	Les M1 – Les Putamen	FC
3	Non-Les M1 – Non-Les SMA	FC	3	Les Thalamus – Non-Les Thalamus	FC	3	Les CST AD	SC
4	Non-Les SMA – Les SMA	FC	4	Age at scan	DE	4	Les M1 – Les Caudate	FC
5	Age at scan	DE	5	Les S1 – Non-Les S1	FC	5	Non-Les M1 – Non-Les SMA	FC
6	ELV	DE	6	Les CST RD	SC	6	Les SMA – Les Thalamus	FC
7	Les Putamen – Non-Les Putamen	FC	7	Les M1 – Les Thalamus	FC	7	Non-Les M1 – Non-Les Thalamus	FC
8	Non-Les M1 – Non-Les Thalamus	FC	8	Non-Les S1 – Non-Les Putamen	FC	8	Les Pallidum – Non-Les Pallidum	FC
			9	Les M1 – Les Caudate	FC	9	Age at scan	DE
			10	Non-Les M1 – Non-Les Pallidum	FC	10	Non-Les SMA – Les SMA	FC
			11	Non-Les CST FA	SC	11	Non-Les CST AD	SC
						12	Non-Les M1 – Non-Les Putamen	FC
						13	ELV	DE
						14	Les S1 – Non-Les S1	FC
						15	Les M1 – Les Pallidum	FC
						16	Non-Les SMA – Non-Les Thalamus	FC
						17	Les CST RD	SC
						18	Les Thalamus – Non-Les Thalamus	FC
						19	Les S1 – Les Putamen	FC
						20	Stroke	DE

Table note: BBT – Box and Blocks Test (BBTA - Affected hand, BBTU – Unaffected hand), AHA – Assisting Hand Assessment, Mod – Modality, FC – Functional connectivity, SC – Structural connectivity, DE - Demographic, Les – Lesioned, CST – Cortical spinal tract, FA – Fractional anisotropy, MD - Mean diffusivity, AD – Axial diffusivity, RD – Radial diffusivity, M1 – Primary motor cortex, S1 – Primary sensory cortex, SMA – Supplementary motor area.

The optimal BBTU prediction model required 11/54 features, which accounted for 11.8% of variance (Table 6). Featuring highly in this model (top 3) was inter-hemispheric functional connectivity within basal ganglia structures (putamen, pallidum) and between lesioned and non-lesioned thalamus. Of the total 11 features, seven involved FC with thalamic and basal ganglia structures. Structural connectivity features accounted for two of eleven features, revealing that microstructure of both lesioned and non-lesioned CST contributed significantly to the BBTU model. Age at scan was also ranked highly (4/11), while none of the other demographic variables (including ELV) were retained in this model.

The optimal AHA prediction model required a much higher number of features with 51/54 used, accounting for 14.2% of variance. This was possibly related to the more complex nature of bimanual tasks. FC between cortical (M1, S1) and subcortical (putamen, caudate, pallidum, thalamus) motor areas ranked highly and composed a large portion of the model (19 of the top 30 features). White matter microstructure (AD) of both the lesioned and non-lesioned CST were also highly ranked (2 of the top 11 features) for predicting AHA performance. Age at scan (ranked 9th of 51), ELV (ranked 13th of 51), and type of stroke (ranked 20th of 51) were retained in the model.

Side of stroke was excluded as not predictive of motor outcomes in all of the BBTA, BBTU, and AHA prediction models and sex was only included in one model. Results from other regression models have been included in supplementary materials.

## 4 Discussion

We have demonstrated that a combination of structural and functional connectivity metrics between cortical and subcortical motor areas predicts clinical motor outcomes after perinatal stroke. Both the lesioned and non-lesioned hemispheres (and the interplay between them) appear to have important roles as do both cortical and subcortical areas. Lesion size, age at scan, sex, and type of stroke were also retained in some of the final models though these often cited clinical features were not highly ranked while side of stroke was not included in any models. Whether unimanual or bimanual motor function was being measured also had strong effects on model structure. Collectively, our results suggest that multimodal imaging models generated with machine learning are likely more capable than single modality approaches of explaining clinical behavior in children with perinatal stroke.

Compared to typically developing peers, children with AIS showed lower functional and structural connectivity for many neuroimaging features, notably the lesioned CST, functional connectivity between cortical regions such as M1, S1 and SMA, as well as inter-hemispheric connectivity within basal ganglia structures. Children with PVI were largely similar to TDC but also showed differences in the lesioned CST and inter-hemispheric connectivity in basal ganglia structures. These different patterns may be due to the relatively larger lesion size in AIS encompassing both subcortical and cortical areas compared to PVI in which injury is more constrained to the periventricular white matter. It is interesting to note that while basal ganglia structures are typically remote from PVI damage, there were still detectible differences in connectivity between these areas compared to peers perhaps mediating motor dysfunction via diaschisis, a process previously investigated in perinatal stroke patients (Finger et al., 2004, Kirton et al., 2016b, Craig et al., 2019a, Craig et al., 2019b, Srivastava et al., 2019). Also of note is the finding that values for some features found to be predictive of motor function in the regression analyses were not different from peers. For example, the non-lesioned CST and multiple cortico-subcortical features (lesioned S1/M1 – lesioned pallidum/caudate/thalamus) were not different between groups but yet were retained by the feature selection algorithm and ranked fairly highly within the regression analyses. This finding suggests that exploring only group differences in neuroimaging markers may not reflect the full richness of compensatory plasticity after very early injury.

The thalamus and basal ganglia structures played a central role in all predictive models. The caudate nucleus, putamen, and pallidum are known to play pivotal roles in the direct and indirect pathways of the motor system, acting as input (caudate and putamen) or output nuclei (pallidum), lesions to which may result in movement disorders in the adult brain (Lanciego et al., 2012). However, although not as well studied, the pattern of motor dysfunction appears to be different when such injuries occur in the perinatal timeframe. Motor outcomes after perinatal stroke have been associated with basal ganglia involvement but only in the extent of hemiparesis severity and spasticity (Boardman et al., 2005, Kirton et al., 2008). The thalamus is also a central hub for integrating sensory inputs shunting information to specialized cortical areas for further processing via significant projections to the basal ganglia and cortex via cortico-basal ganglia-thalamo-cortical loops (Lanciego et al., 2012). We have recently demonstrated that thalamic diaschisis is common in perinatal stroke (Srivastava et al., 2019) and that altered thalamic volumes, particularly in the non-lesioned hemisphere, are associated with motor function (Craig et al., 2019a). Our findings here appear consistent with this previous evidence supporting an important role for the basal ganglia and thalamus in the developmental plasticity that determines motor function after perinatal stroke.

Another highly ranked feature in the predictive models was the inter-hemispheric functional connectivity between the lesioned and non-lesioned supplementary motor areas (SMA). We also found a highly ranked contribution for FC between non-lesioned M1 and non-lesioned SMA in multiple models. It has previously been suggested that the SMA may play a role in compensation and motor reorganization after stroke. Motor task fMRI studies in well-recovered adults post-stroke have found more extensive SMA activations (Nair et al., 2007) compared to controls that correlated with function (Rehme et al., 2012). We have also shown evidence of topographical re-organization of task-related SMA activations in children after perinatal AIS but did not find associated differences in activation strength, extent, or correlation with motor function (Baker et al., 2020). In children with PVI, we have also demonstrated correlations between inter-hemispheric SMA FC at rest and performance on specialized sensory tasks using a proprioceptive robot (Woodward et al., 2019). Given that the SMA areas lie relatively anterior and on the medial surface of the brain, they are often spared after middle cerebral artery infarction, and SMA recruitment may be an ideal compensatory mechanism after stroke. After PVI, damage is often limited to periventricular white matter and the SMA is typically preserved. Further, in intact brains, SMA is purported to make up ~ 10% of the connections in the CST, directly synapsing with motor neurons (Nachev et al., 2008), suggesting the enhanced retention of such upper motor connections that are prominent at the time of perinatal stroke. Our findings that the FC of SMA is a highly ranked feature in multiple motor outcome models is consistent with evidence of SMA’s central role in re-organized motor systems after stroke and could be explored as a possible accessible target for neuromodulation.

We also found that connectivity of both the lesioned and non-lesioned hemispheres was highly ranked for most motor function predictions. Even though the BBTA is a unimanual task quantifying function of the weaker, stroke-affected hand, the regression models included features from the lesioned and non-lesioned hemisphere as well as features integrating both hemispheres via inter-hemispheric connectivity. Similarly, for the bimanual AHA model, many highly ranked features involved the lesioned and non-lesioned hemispheres, as well as features involving bilateral inter-hemispheric connectivity. These findings are consistent with the well-established, essential role that the non-lesioned hemisphere plays in determining motor function in many children with perinatal stroke (Eyre, 2007, Staudt, 2007, Hilderley et al., 2019). Evidence from multiple modalities including task fMRI and transcranial magnetic stimulation (TMS) studies demonstrates that the majority of children with perinatal stroke have both ipsilesional and contralesional corticospinal projections to the affected hand (Thickbroom et al., 2001, Staudt et al., 2002, Vandermeeren et al., 2003, Guzzetta et al., 2007, Dinomais et al., 2013, Van de Winckel et al., 2013, Arichi et al., 2014, van der Hoorn et al., 2014, Zewdie et al., 2017, Weinstein et al., 2018). Maintaining functional control in ipsilesional areas has been associated with better clinical motor function as compared to prominent recruitment of contralesional areas although considerable variability exists (Chu et al., 2000, Staudt et al., 2002, Weinstein et al., 2014, Zewdie et al., 2017). Combining this substantial evidence base with our findings in the current study suggests that future models capable of incorporating diverse metrics beyond just neuroimaging that also consider the roles of both hemispheres may be even better at revealing the determinants of clinical function.

Interestingly, bilateral sensorimotor connectivity, one of the most previously studied networks in this population, was not consistently retained in some models though interhemispheric sensory (S1-S1) connectivity was highly ranked for the PVI BBTA and AHA models. Involvement of intra-cortical motor (M1) and sensory (S1) areas was not as highly ranked as we had expected for AIS. Inter-hemispheric FC between bilateral M1 areas was also not retained in any model. Connectivity between S1 and M1 were either ranked as quite low or not included. This could be because the AHA and BBTA tasks have both sensory and motor components, making it difficult to tease out the relative contributions of each component unless utilizing specialized, purely sensory tasks such as those performed previously using robotics (Kuczynski et al., 2016, Kuczynski et al., 2017, Woodward et al., 2019). Another possibility is that contributions of motor and sensory areas were highly correlated (*i.e.*, redundant) with each other or other variables that were therefore reduced in some models. It is plausible that fluctuations in BOLD response (*i.e.*, FC) between M1 and S1 areas were highly correlated with each other (Saunders et al., 2019) or that the tractography algorithms isolating corticospinal tracts also included the sensory (dorsal column medial lemniscus) tracts (Kuczynski et al., 2017), and therefore were excluded by the RELIEFF feature ranking and selection algorithm. Although important, cortical motor and sensory FC may not have contributed unique information to the model over and above other features and were therefore discarded in favor of more predictive cortical-subcortical connectivity metrics. As the first study of its kind to incorporate all such networks simultaneously in this population, it is possible we have been over-estimating the importance of the cortical sensorimotor structures we had greater ability to interrogate at the expense of the deeper network components, the importance of which may be substantial.

In perinatal stroke, age at scan can also be considered a proxy for time since stroke reflecting not just placement on the developmental trajectory, but also the duration of possible neuroplastic re-organization. Age at scan was considered to be a predictive feature in some models but not the AIS prediction models. This finding could reflect that older children have had more time to engage compensatory mechanisms and recruit additional brain areas to preserve motor function even though all children were in the chronic stage post-stroke. This could also reflect differences between AIS and PVI in when the injury occurs in the perinatal period.

Interestingly, lesion size (*i.e.,* ELV) was not as highly ranked as expected. We have informally observed in the past that the size of the lesion may be less important as compared to location in predicting motor outcomes from perinatal stroke. This is consistent with previous studies where lesion volumes have often been included but associations with outcomes have been modest (Mackay et al., 2020). Certainly, some children with very large strokes do quite well and others with relatively small lesions can have profound motor impairments. Our current findings provide quantitative evidence that size of lesion is not highly predictive of motor function though it does play a moderate role. Conversely, side of stroke was not associated with motor outcomes and sex did not figure prominently in prediction models. Left-side arterial ischemic strokes are more common than right-side strokes though the reason for this is poorly understood (Dunbar and Kirton, 2018). This incidence was mirrored in our sample having more children with left strokes (~60%) than right (~40%). Furthermore, the finding that sex did not significantly contribute to motor outcomes models is consistent with male and female children having similar outcomes despite perinatal stroke being more common in males (Dunbar and Kirton, 2018).

That the BBTA, BBTU and AHA models required different numbers of features for optimal prediction likely relates to the more complex and naturalistic nature of the bimanual tasks reflected in the AHA. For the AIS group alone, only two features were required to optimally predict AHA performance compared to 28/54 features for the PVI group. Additional neuroimaging features that could be added to improve model accuracy include morphometric measurements of additional cortical and subcortical structures [including volumetrics, cortical thickness, gyrification and sulcal geometry (Dubois et al., 2008, Pagnozzi et al., 2016, Al Harrach et al., 2019)], more specific quantifications of white matter myelination (Glasser and Van Essen, 2011, Ganzetti et al., 2014), neurometabolite features from MR spectroscopy studies (Carlson et al., 2017), and blood perfusion metrics from arterial spin labelling studies (Wintermark and Warfield, 2012, De Vis et al., 2013, Watson et al., 2016). In addition, cognitive variables could also be considered here since the three tasks we used could be affected by a child’s overall processing speed, comprehension of instructions, attention, compliance, and other complex factors (Fuentes et al., 2016). A major advantage of many machine learning regression models is no assumptions regarding normality of statistical distributions or non-collinearity among variables are required. Thus, these methods will be powerful techniques with which to move forward the field of neuroimaging and patient-centered precision medicine.

This study may provide additional evidence informing theoretical models of neuroplasticity after early injury. Both cortical and subcortical inter-hemispheric functional connectivity was rated consistently highly in the regression models suggesting that both lesioned and non-lesioned hemispheres likely interact after stroke to compensate for injury where possible. This is consistent with evidence from task-fMRI (Chu et al., 2000, Staudt et al., 2002, Weinstein et al., 2014) and TMS (Staudt, 2010, Zewdie et al., 2017) literatures that maintaining function in perilesional areas results in better function and that restoring inter-hemispheric excitatory and inhibitory balance (Ferbert et al., 1992) via non-invasive brain stimulation may be an effective intervention (Kirton et al., 2016a, Kirton et al., 2017, Eng et al., 2018, Hilderley et al., 2019). Whether inter- or intra-hemispheric connectivity is more important for models of neuroplasticity is nonetheless still unclear. Our results suggest that features measuring *both* inter- and intra-hemispheric connectivity are predictive of motor function and that restoring balance to the motor network may underlie functional recovery. No doubt a considerable amount of among-patient variability also exists. Future developments of neuroplasticity models should also more explicitly include subcortical thalamic and basal ganglia structures given their relatively large role in these predictive regression models for both unimanual and bimanual function.

We acknowledge several limitations. First, our patient sample included only those children who were older (>6 years) and relatively less impaired on average given the need to complete a complex MRI study. This bias is common in imaging studies, however may limit the generalizability of these results to very young or more profoundly disabled perinatal stroke populations. Normalization procedures often under-perform when used in the presence of lesions (Andersen et al., 2010, Ripollés et al., 2012). While we ensured the accuracy of the segmentation and normalization processes by examining each scan slice-by-slice, and excluded one patient who had a large lesion that could not be accurately segmented, we concede that standard normalization procedures are not ideal for use in stroke brains. More specialized procedures such as DARTEL (Ashburner, 2007) or Symmetrical Normalization (SyN) (Avants et al., 2008) may be better suited to solve this challenge (Klein et al., 2009). Cost-function masking, although time-consuming, has also been shown to be effective in the presence of stroke lesions (François et al., 2016, François et al., 2019) as has the use of pediatric templates for normalization (Fonov et al., 2011, Richards et al., 2016). Our use of the ELV metric had the advantage of quantifying lesion size in a manner that was directly comparable in patients with different lesion etiologies (AIS vs PVI), however may have been relatively insensitive to very small lesions or to those with periventricular cysts accompanied by small dilatations in ventricle size. We also included a larger number of functional connectivity (41/54) compared to structural connectivity (8/54) features. Of the structural features included, all were unilateral (*i.e.*, lesioned and non-lesioned CST) and did not include inter-hemispheric structural connectivity (*i.e.*, motor fibres of the corpus callosum). Our clinical outcome measures (BBTA, BBTU, AHA) were primarily motor in nature, however a direct investigation of connectivity using a purely sensory task or other behavioural measures may further inform more diverse disabilities after perinatal stroke. Further, the amount of rehabilitation and physiotherapy given throughout early childhood is likely an additional predictor of motor function that was not available for inclusion here. The use of an index reflecting prior therapy dosing may have made the predictive power of the regression models better since children likely vary widely on this metric and we would encourage future studies to include this information.

## 5 Conclusions

We have demonstrated in a group of children with perinatal stroke that neuroimaging features capturing structural and functional connectivity can be used for individual, data-driven prediction of motor function. Cortical and sub-cortical connectivity across both hemispheres are important predictors of clinical motor function, the degree of which depends on which motor function (unimanual versus bimanual) is being measured. Machine learning regression models are a powerful new tool to advance our understanding of developmental neuroplasticity in children with early brain injury and may inform personalized targets for neuromodulation.

## CRediT authorship contribution statement

**Helen L. Carlson:** Conceptualization, Methodology, Formal analysis, Visualization, Writing - original draft, Writing - review & editing. **Brandon T. Craig:** Software, Data curation, Formal analysis, Writing - review & editing. **Alicia Hilderley:** Software, Data curation, Formal analysis, Writing - review & editing. **Jacquie Hodge:** Formal analysis, Writing - review & editing. **Deepthi Rajashekar:** Software, Data curation, Formal analysis, Writing - review & editing. **Pauline Mouches:** Software, Data curation, Formal analysis, Writing - review & editing. **Nils D. Forkert:** Conceptualization, Methodology, Formal analysis, Writing - review & editing. **Adam Kirton:** Conceptualization, Methodology, Supervision, Funding acquisition, Writing - review & editing.

## Declaration of Competing Interest

The authors declare that they have no known competing financial interests or personal relationships that could have appeared to influence the work reported in this paper.
